# Development and evaluation of deep neural networks for the classification of subtypes of renal cell carcinoma from kidney histopathology images

**DOI:** 10.1038/s41598-025-10712-9

**Published:** 2025-08-05

**Authors:** Amit Kumar Chanchal, Shyam Lal, Shilpa Suresh

**Affiliations:** 1 School of Computing, MIT Vishwaprayag University, Solapur, Maharashtra 413255 India; 2 Department of Electronics and Communication Engineering, National Institute of Technology Karnataka, Surathkal, Mangaluru, Karnataka 575025 India; 3 Department of Mechatronics, Manipal Institute of Technology, Manipal Academy of Higher Education, Manipal, Karnataka 576104 India

**Keywords:** Cancer, Computational biology and bioinformatics, Health care, Oncology

## Abstract

Kidney cancer is a leading cause of cancer-related mortality, with renal cell carcinoma (RCC) being the most prevalent form, accounting for 80–85% of all renal tumors. Traditional diagnosis of kidney cancer requires manual examination and analysis of histopathology images, which is time-consuming, error-prone, and depends on the pathologist’s expertise. Recently, deep learning algorithms have gained significant attention in histopathology image analysis. In this study, we developed an efficient and robust deep learning architecture called RenalNet for the classification of subtypes of RCC from kidney histopathology images. The RenalNet is designed to capture cross-channel and inter-spatial features at three different scales simultaneously and combine them together. Cross-channel features refer to the relationships and dependencies between different data channels, while inter-spatial features refer to patterns within small spatial regions. The architecture contains a CNN module called multiple channel residual transformation (MCRT), to focus on the most relevant morphological features of RCC by fusing the information from multiple paths. Further, to improve the network’s representation power, a CNN module called Group Convolutional Deep Localization (GCDL) has been introduced, which effectively integrates three different feature descriptors. As a part of this study, we also introduced a novel benchmark dataset for the classification of subtypes of RCC from kidney histopathology images. We obtained digital hematoxylin and eosin (H&E) stained WSIs from The Cancer Genome Atlas (TCGA) and acquired region of interest (ROIs) under the supervision of experienced pathologists resulted in the creation of patches. To demonstrate that the proposed model is generalized and independent of the dataset, it has experimented on three well-known datasets. Compared to the best-performing state-of-the-art model, RenalNet achieves accuracies of 91.67%, 97.14%, and 97.24% on three different datasets. Additionally, the proposed method significantly reduces the number of parameters and FLOPs, demonstrating computationally efficient with 2.71 × $10^9$ FLOPs & 0.2131 × $10^6$ parameters.

## Introduction

Kidney cancer is one of the increasing diseases worldwide in both males and females. Renal cell carcinoma (RCC) is the most common kidney cancer^1,2^ that accounts for 2–3% of adult cancers and constitutes approximately 85% of all primary renal malignant tumors. Genetic factors, tobacco exposure, obesity, and hypertension are the most significant and generally accepted risk factor for kidney cancer. The major histologic class of RCC^3^ include clear cell RCC (KIRC), papillary cell RCC (KIRP), and chromophobe RCC (KICH) accounting for (70–80%), (14–17%), and (4–8%) of RCC respectively. The classification of RCC data is based on morphology and cytogenetic characteristics. Each of these class has different prognoses and differs in cytogenetic and histopathologic profiles. Clear cell RCC is the most common subtype of cancer in adults. Cells of clear cell RCC have clear cytoplasm and have spherical masses that distort the renal outline. Tumors of clear cell RCC are composed of large uniform cells with abundant cytoplasm that is rich in glycogen. Hereditary syndromes related to clear cell RCC is von hippel lindau (VHL) and mutations in the VHL gene lead to the development of tumors. Papillary RCC is the second most common renal cancer and is characterized by prominent papillary structures lined by monomorphic cuboidal epithelial cells and cells with abundant eosinophilic cytoplasm. Tumor cells in KIRC have a rounded or polygonal shape while KIRP is of cuboidal structure and low columnar cells are arranged like papillary formations. Chromophobe RCC is characterized by pale cells and smaller eosinophilic tumor cells in variable proportions with wrinkled nuclei and perinuclear halos. Cell membranes of KICH appear very distinct with prominent margins.

Currently, the clinical examination steps to diagnose cancer disease usually begins with basic imaging tests like CT scans, magnetic resonance imaging (MRI), nuclear scan, and others. If the doctor suspects any unusual growth through these tests then the next step is a biopsy. A biopsy is a procedure in which a doctor removes a sample of diseased tissue and a pathologist looks at this tissue under a microscope and decides whether the tissue is cancerous or not. Digital histopathological images are obtained from the microscopic examination of the stained biopsy tissues. Once it is confirmed that the tissue is cancerous then they need to do some more tests to figure out the stage and grade of cancer. Identification of stage and grade is very important to plan further treatment. A pathologist report affirmed that the stage of diagnosis is the most important prognostic factor. In these cases, continuous staging evaluation is extremely important for the clinical management of patients. The above process is time-consuming, prone to human error, and highly depends on the expertise of a pathologist. Early detection and classification of kidney cancer tissues enable doctors and practitioners to decide the further course of treatment. Therefore, fast and precise analysis of kidney cancer tissue images is extremely important for proper diagnosis. With the exponential growth of cancer cases, the computer-assisted diagnosis (CAD) of histopathology images has been used in preference and replaced the manual diagnosis for fast and precise assessment. Here we reviewed the state-of-the-art deep learning solution for the classification and grading of histopathological images, techniques to extract relevant information from the digital histopathologic patches, and techniques to retain the extracted information.

### Deep learning solutions for classification of H & E stained images

The histological structure of clear cell RCC primarily has eosinophilic cells with thin-walled, staghorn-shaped vasculature. Papillary RCC has prominent papillary structures and foamy macrophages, while Chromophobe RCC consists of a prominent cell membrane, irregular nuclei, perinuclear halo, and pale to eosinophilic cytoplasm. With the use of the CNN model^4^, the inherent features of renal tumors can be identified by capturing their unique characteristics. The automatic classification framework^5,6^ of RCC employed CNN pre-trained on ImageNet, and for grading,^7^ used the Lasso model which includes a certain number of features to predict the grade. The works^8–13^ where deep learning is successfully applied for the detection of malignant tumor cells in breast tissues. The study^14^ used a highly optimized CNN module and not using any pre-trained weights is very effective in tumor detection of BreaKHis data. The study of Hepatocellular carcinoma (HCC) in liver tissue^15,16^ used transfer learning, while^17^ CNN model trained from scratch on the TCGA liver dataset. In this work, we have studied both transfer learning as well as the CNN model trained from scratch. The proposed model does not use any pre-trained weights and effectively works on multiple organ histopathology datasets.

### Techniques to extract relevant information

Attention mechanisms is widely employed in the field of segmentation and classification of histopathology images to focus on relevant features. Attention-based feature descriptor^18–21^ where two different attention modules called channel attention and spatial attention are simultaneously incorporated to assign different weights on objects of different scales. The work^22^ generates high-resolution spatial maps by attention pooling to predict class from X-ray images. A naive stacking of the attention mechanism leads to a performance drop in the network. The new residual attention mechanisms^23^ solved the problem by incorporating techniques like stacked network structures, and a mixed attention mechanism. The representative works^24,25^ deployed attention module in the network and focused to avoid local constraints and aggregates global context. The behavior of channel-wise discriminative features adaptively calibrated in squeeze-and-excitation^26,27^ to model the interdependences between channels. BHCNet^14^ demonstrated the use of attention mechanism in histopathology image classification by using a small ResNet architecture along with squeeze and excitation block for binary and eight class classification of BreakHis dataset with less computational complexity. BreastNet^11^ contains channel attention, spatial attention, residual unit, and hypercolumn unit to achieve better classification accuracy. LiverNet^17^ for four-class classification of liver hepatocellular carcinoma from liver histopathology images, incorporated attention modules and atrous spatial pyramid pooling block to capture the representative feature. In this work, we adopt a transformation method based on squeeze and excitation (SE), a lightweight gating mechanism that helps to obtain improved performance.

### Techniques to retain extracted features

**Fig. 1:**
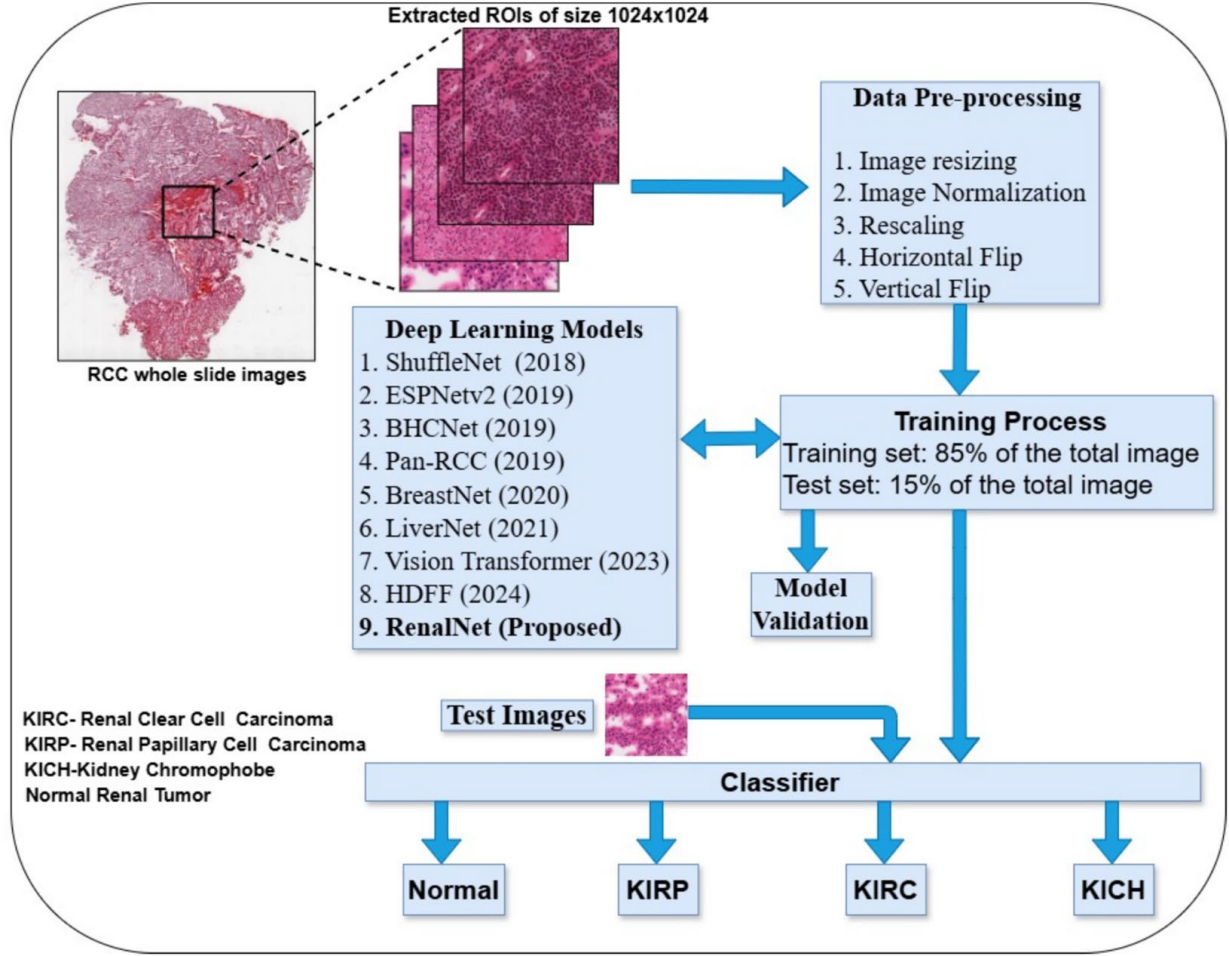
Classification pipeline of proposed RenalNet.

Many works retain spatial information by incorporating skip connections^28,29^ to avoid degradation of information between output and input and utilized all preceding layers as input to the next layer throughout the network. This idea is effective in feature reuse and thus reduces the vanishing gradient problem and therefore training of a large number of deep neural network layers became simpler. CNN models^30,31^ were proved to capture more complex features as the model depth increases, and by increasing width, models can capture fine-grained features. Two-layer shallow model and a twenty-two-layer deep network^32^ were tested on the TCGA kidney dataset and concluded that the deep model classified low-grade granular tumor and high-grade clear cell carcinoma better than the shallow model. Resolution is another factor that improves accuracy but was observed to saturate and adding more layers further leads to higher training errors. Parallel layers of convolution modules^33–35^ showed that increased cardinality works better than going deeper or wider in the network and adopts residual connection to concatenate multiple paths of a set of transformations. Although these methods are effective, the above techniques used the traditional transformation method to retain the extracted features. In this work, we propose a CNN model that effectively enriches nuclear features by fusing the information of multiple parallel branches and attains a considerable margin on the existing network. Recent advancements in RCC subtyping and grading offer new insights and opportunities. The integration of heatmaps for tumor grade identification^36^ has proven valuable. Techniques such as ExpertDT^37^ CNNs, combined with pathologist’s expertise, have further refined RCC classification processes. Chen et al.^38^ proposed a general-purpose self-supervised model pretrained on 100 million images, demonstrating the potential of large-scale data pretraining in medical imaging. Additionally, hybrid multi-instance model^39^ incorporating transformers and graph attention networks have shown promise in capturing complex tumor characteristics. The use of vision transformers^40,41^ for RCC subtyping represents a cutting-edge deep learning architecture advancing histopathology analysis. Hybrid Deep Feature Fusion (HDFF) technique^42^, combines feature vectors from EfficientNetB3 and ResNet50, provides a more comprehensive and robust representation of histopathology images. An extremely efficient convolutional neural network designed for edge devices^43^, along with a general-purpose lightweight and power-efficient CNN model^44,45^ has been included in our study. In previous works, deep learning approaches for the classification of histopathology images were focused on transfer learning^5,8,10,15,16,38^ techniques. Recently, a pre-trained model^5^ used ImageNet dataset weights with directed acyclic graphs and SVM on top of it for the classification of three sub-types of RCC of the TCGA dataset. Another method for five class-classification of RCC cases^6^ used ResNet-18, trained and validated with multiple datasets. In^7^, Clear cell carcinoma grading is performed using the Lasso model with TCGA clear cell RCC dataset. Most of the datasets used for this purpose have an issue of high-class imbalance and there is no publicly accessible processed data for RCC classification available for the reproducibility of the result. Transfer learning effectively transfers powerful texture features; however, different RCC classes’ microscopic structures and clinical features extend beyond texture alone. This leads to the requirement of deep learning models which is capable of extracting highly variable nuclear features from RCC data. The potential and applicability of deep learning models for the classification of histopathology images have been demonstrated by many researchers but reported results are not optimal for clinical use. Variants of RCC typically have distinct biological and clinical behavior, varying morphological patterns, and therapeutic responses. Different morphological patterns of nuclei and distinguishable intra-nuclear features at the microscopic level provide the foundation for the development of a convolutional neural network (CNN) model. This study focuses to demonstrate a fast and accurate deep-learning technique for the classification of RCC kidney H&E stained histopathology images. The methodology that has been used for training and prediction can be observed in Fig. 1. We acquired digital hematoxylin and eosin-stained WSIs of 950 different cases from The Cancer Genome Atlas (TCGA), a benchmark database resource. To balance the dataset we have considered 679 cases out of 950 cases, where morphological and clinical features are highly relevant and expressive. All extracted patches are of size (1024 × 1024) and each of the four classes namely Normal, KIRC, KIRP, and KICH have an equal number of ROIs.

Our study is different from others for several reasons: (1) Of course, the transfer learning approach transfers the powerful feature, the development of an end-to-end deep learning model that is intended to capture morphological and clinical features as well as overall tissue organization for the classification of RCC classes from kidney histopathological images have not been focused on. (2) The ultimate aim of using AI in a clinical workflow is speed and accuracy. The proposed method meets both requirements. (3) The introduced dataset is class balanced compared to any other RCC dataset. (4) We performed comparable experiments on multiple organ histopathology data to verify the validity of our proposed method. (5) We have also included the performance of networks leveraged by transfer learning of pre-trained ImageNet weights. To address the challenges in classifying RCC kidney histopathological images, our contributions are as follows:

1. With the close collaboration of an experienced healthcare team and deep learning/AI professional, we proposed an efficient and most accurate end-to-end deep learning architecture called RenalNet for the classification of RCC subtypes from kidney H&E stained histopathology data.
2. This study proposes a CNN module called multiple channel residual transformation (MCRT) to focus on the most relevant morphological features of RCC. Another novel module called group convolution deep localization (GCDL) effectively integrates three different feature descriptors. These blocks contribute maximally to the proposed RenalNet model and complete the classification process.
3. A new benchmark kidney histopathology dataset for the classification of renal cell carcinoma is developed from the WSIs of TCGA data and will be made available for the reproducibility of the result.
4. The proposed RenalNet requires minimum computational resources, less time to train, and outperforms the eight most recent benchmark models on multiple organ histopathology datasets.

The paper follows this structure: The ”Methods” section introduces the novel dataset, outlines the training process, and describes the implementation of the proposed model. In the ”Results and Discussion” section, the performance of the proposed model is compared with other state-of-the-art classification techniques. We also analyzes the impact of key components of the proposed network through an ablation study, statistical analysis, and computational complexity.

## Methods

### Dataset and image processing

The introduced kidney histopathology dataset contains normal renal tissue (Non-cancerous), Renal Clear Cell Carcinoma (KIRC), Renal Papillary Cell Carcinoma (KIRP), and Kidney Chromophobe (KICH) histopathology images of the Renal Cell Carcinoma (RCC). However, not all four classes contributed equally to the dataset. The KICH class had the minimum contribution, and we were able to extract 1100 patches of size 1024x1024 pixels from it. Based on the number of ROIs obtained in the KICH class, we extracted the same number of ROIs from the other classes. Out of 950 cases we have considered 679 cases, where morphological and clinical features are highly relevant and expressive. This approach ensured that the introdued dataset has an identical number of ROIs for each class, with all classes contributing equally to the dataset. Table 1 provides a detailed view of tissue pattern and the overall organization of RCC classes and Normal tissue regions. We computed and analyzed our experimental results on the patches created from the WSI of TCGA. The patches creation phase was carried out under the supervision of experienced pathologists who perform routine diagnoses. We cropped only those parts of WSI that have histologically defined clinical features. A patch was considered to be cancerous if the majority of the area had an abnormal region. The created TCGA kidney dataset has a total of 4400 patches belonging to four classes of kidney tissue extracted from 679 different cases. All extracted patches are of size (1024x1024) and each of the four classes namely Normal, KIRC, KIRP, and KICH have an equal number of ROIs. The processed data from WSI of TCGA used in^5,6^ is not publicly accessible and there exists a high-class imbalance. It will not be a fair assessment of a deep learning network if, in multi-class classification, one class contains the majority of the total data. However, the newly introduced dataset is class-balanced and will be made accessible for the reproducibility of the results. We split the dataset before any data augmentation to avoid overfitting issues and obtained 4400 patches that were slidewise distributed into training (85%), and testing (15%). The validation set was created by randomly selecting patches from the training set. All the images were resized to 224x224 and normalized to zero mean and unit variance before passing them to deep learning architecture for training. Results are evaluated on 632 test images. The testing phase was performed by the patches of different cases and it is from those WSI which was completely unseen for the model. Table 2 gives the complete overview of the data collection process, including the number of cases and the number of images obtained per class. To verify the validity of our proposed method, we have used two additional liver histopathology datasets^17^ obtained from Kasturba Medical College (KMC), Mangalore, MAHE, Manipal, Karnataka, India.

**Table 1:** Histology and clinical features of RCC subtypes and normal tissue regions at 40x resolution.

Clear cell RCC	Papillary cell RCC	Chromophobe RCC	Normal
			
Clear cells with thin-walled	Prominent papillary structures lined by monomorphic cuboidal epithelial cells.	Large eosinophilic vegetal-like cells, in finely granular cytoplasm.	The cells of the proximal tubules have central nuclei and very acidophilic cytoplasm.
Few cells are eosinophilic and granular.	Bilateral and multiple masses.	Smaller eosinophilic tumor cells are in variable proportions with wrinkled nuclei	Cells show prominent vacuoles.
Tumor shows large uniform cells with abundant cytoplasm that is rich in glycogen.	Finger-like projections with fibro-vascular cores.	Cell membranes of KICH appear very distinct with prominent margins.	Each renal corpuscle has a vascular pole and a tubular pole.
Cells are organized in acinar, alveolar, and tubular form.	Papillary carcinoma is multi-focal in origin.	Nuclear grade does not apply.	The renal cortex on microscopy reveals glomeruli and tubeless.
Well-defined margins and confined to the renal capsule.	Surrounded by a fibrous capsule.	Typically solid with parenchymal extension.	A normal glomerulus by light microscopy shows glomerular capillary loops which are thin and delicate.
Tumors in KIRC are composed of cells that have clear cytoplasm and have spherical masses.	Type-1 Cells form a single layer on papillae	Irregular nuclei.	Endothelial and mesangial cells are normal in number.
Staghorn-shaped vasculature.	Type-1 Composed of small cells having scanty basophilic or pale cytoplasm	Perinuclear clearing	The surrounding tubules are lined by cuboidal epithelium.
Tumors have delicate branching fibrovascular septae.	Type-2 is of large cells with abundant acidophilic cytoplasm.	Pale to eosinophilic cytoplasm.	
		Arranged in solid sheets.

**Table 2:** Datasets class distribution at 40X.

New introduced	Total cases		Extracted ROIs (1024x1024)		Tabibu et al.^5^	
Dataset	Training	Test	Training	Test	Total extracted ROIs:Training+Test	
Normal	164	29	942	158	–	
KIRC	157	28	942	158	1027	
KIRP	149	26	942	158	303	
KICH	107	19	942	158	254	
	**Total Cases=679**		**Total ROIs=4400**		**Total ROIs=1584** **(Variable Size)**	

Significant values are in bold.

The whole slide images and clinical information were downloaded from TCGA data portal (https://gdc.cancer.gov/). The conducted research reported in this article involving human participants was in accordance with the ethical standards of the institutional and/or national research committee and with the 1964 Helsinki declaration and its later amendments or comparable ethical standards.

### Training & Implementation

 Training and implementation of the proposed RenalNet architecture were conducted on Dell-G4G3NSM workstation with 8 GB NVIDIA QUADRO P4000 GPU. We have used TensorFlow 2.4.1, Python-3 environment to speed up the entire process^46–48^. We trained our model on the training set for a given range of hyperparameters and evaluated the efficiency of the model on the test and validation datasets. After creating patches of size (1024 × 1024 × 3) from the whole slide image, these patches were resized to (224 × 224 × 3) and given as input to the proposed RenalNet model. We trained the model with Adam optimizer^49^, with a default learning rate (0.001). Categorical-Crossentropy^11,17^ is used to optimize the loss and the softmax classifier^14^ at the final layer completes the classification process. These are the important controlling hyperparameters that we tuned: (1) Reduce learning rate of factor = 0.5, Patience = 5, Min-delta = 0.0001, and monitor the validation accuracy. (2) Batch size = 4 (3) The size of the convolution filter = 16, 32, and 64. (4) Dilation rates = 1,2,4,6 (5) Total Epoch = 65. To prove the worthiness and efficacy of the proposed RenalNet model, we carefully trained and tested all models for three trials on the TCGA kidney dataset. We calculated the final results based on an average of three trials by initializing random weights in each trial. Class-wise and the overall performance matrices of the model were measured to analyze the performance of the network. The important controlling hyperparameter that we tuned is shown in Table 3. Performance evaluation matrices used in this study are precision, recall/sensitivity, F1-Score, and *Accuracy*. *Precision*^5,11^ focuses to minimize mistakes in guessing positive labels while *recall*^17^ measures how well positives are recalled. *F1-score*^6^ maximizes both recall and precision by calculating the harmonic mean between recall and precision. *Accuracy* measures the ratio of right predictions to the total number of predictions made. The data and code are available at https://github.com/chanchalnitk/RCC_Classification

**Table 3:** Details of hyperparameters.

Optimizer	Adam with initial learning rate of 0.001
Batch size	4
Convolution filter	16, 32, 64
Dilation rates	1,2,4,6
Total epochs	65
Reduce learning rate	Factor=0.5, patience=5, min-delta=0.0001, Monitor-Validation acc.
Early stop	Patience=30, min-delta=0.0001, Monitor-Validation acc., restore-best-weights

### Proposed architecture

**Fig. 2:**
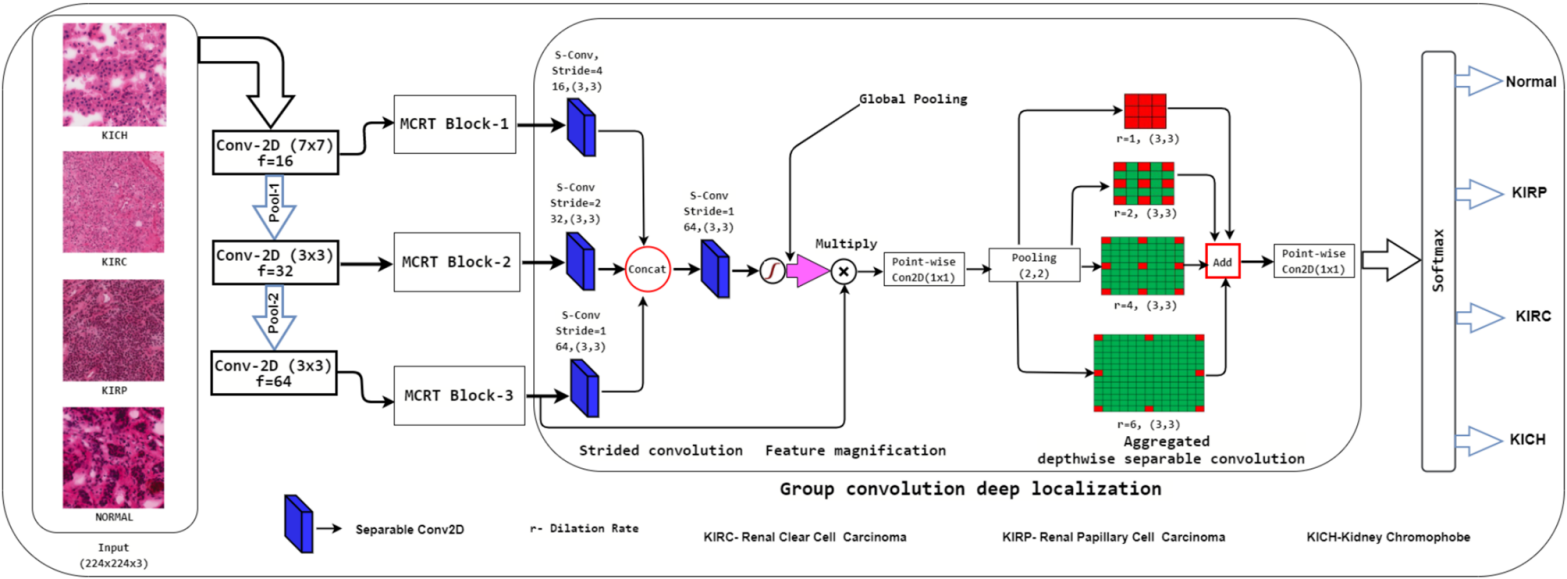
RenalNet model for classifying RCC data from H&E-stained histopathology images.

The RenalNet model comprises four important phases which are (1) Data preparation (2) Multiple channel residual transformation (3) Group convolution deep localization network (4) Finally classification phase, where the model works on patches of whole slide images of TCGA dataset. The data preparation phase is carried out under the supervision of experienced pathologists and results of the proposed RenalNet are computed on the patches created from the WSI of TCGA. The RenalNet is designed to capture cross-channel and inter-spatial features at three different scales simultaneously and combine them together. Cross-channel features refer to the relationships and dependencies between different data channels, while inter-spatial features refer to patterns within small spatial regions. To capture cross-channel features, squeeze-and-excitation principles^10^, channel attention mechanisms^18^, and depthwise separable convolutions^44^ are utilized, while spatial attention mechanisms, dilated (Atrous) convolutions, and fully convolutional networks (FCNs) are employed to extract inter-spatial features. The multiple channel residual transformation (MCRT) enables the model to extract cross-channel features while focusing on the main subject of the input data. To capture and extract a more representative set of features, we employed a Group Convolution Deep Localization (GCDL) network. The GCDL integrates three feature descriptors: strided convolution, attention mechanisms, and an aggregated depthwise separable convolution block This integration allows the network to generalize and adapt effectively to a variety of data. The structure of the proposed RenalNet model for the classification of RCC data is shown in Fig. 2.

MCRT is composed of the convolutional unit, squeeze, and excitation (SE) channel attention unit, and one identity connection. MCRT focus on two aspects: First, it achieves multiple equivalent receptive fields by utilizing different squeeze ratio, which effectively enriches the nuclear features at three different points within the network, and second, multiple parallel branches produce very fine spatial maps at very low computational complexity. The standard convolution operation in three layers of MCRT operates on both spatial and channel-wise features within local receptive fields. SE channel attention unit^26^ in deep learning is a mechanism that focuses on the main subject of input data for some layers to create corresponding output by concentrating only on the relevant features. SE block re-calibrates the channel-wise feature by producing a channel descriptor across its spatial span. To generate channel-wise statistics, the first step of the SE block is to squeeze the global spatial information into the channel parameters called channel descriptors. Here the input feature map is first converted into a vector using a global average pooling operation. A dense layer with ReLU activation squeezes the layer to different ratios. Since the channel attention mechanism concentrates on the most relevant channels^26–28^. By using three different ratios we got channel depth of *f*, *f*/2, and *f*/4. By fusing the outputs of three SE blocks with different channel ratios, it is possible to further strengthen by capturing the most relevant content contributed by different channel depths.

**Fig. 3:**
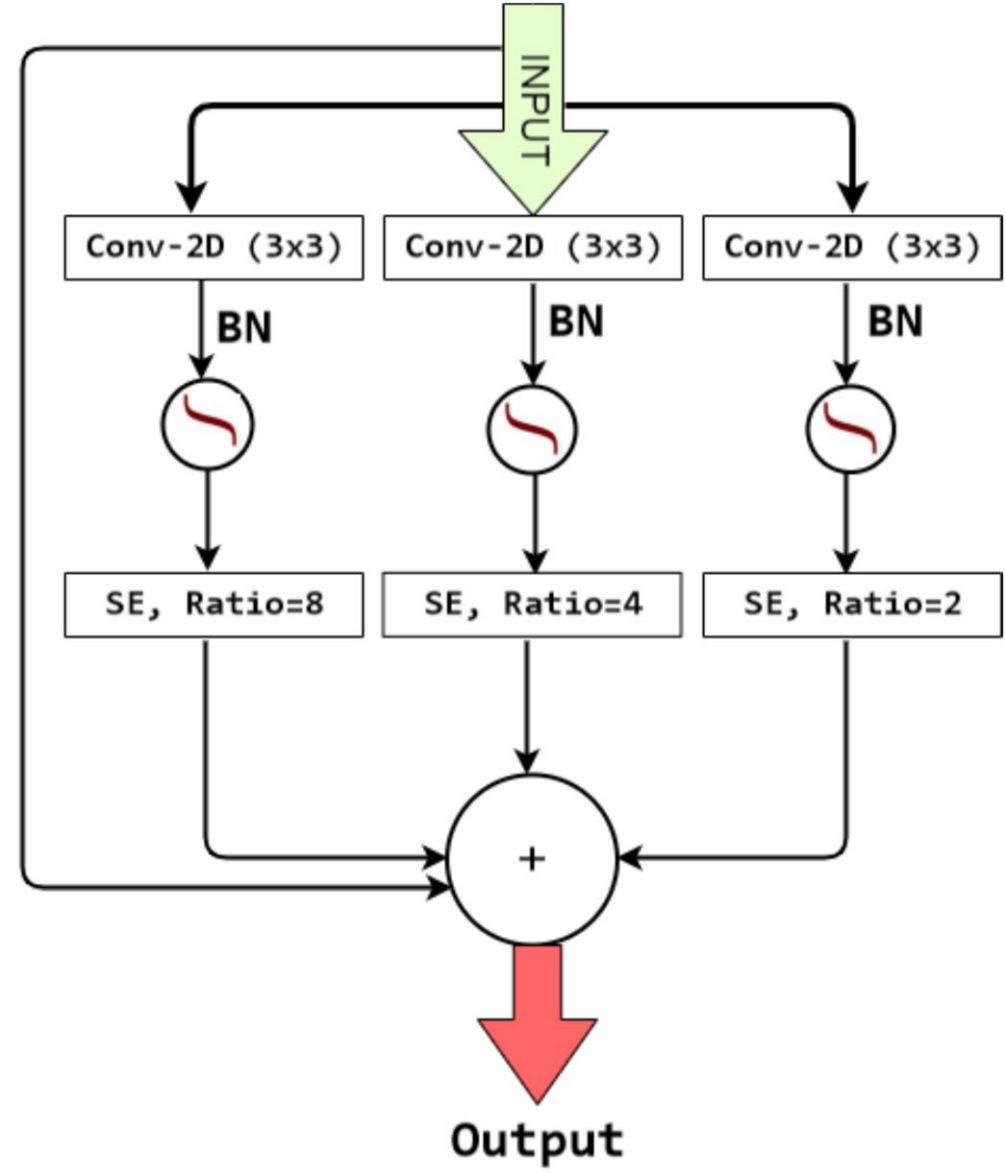
Multiple channel residual transformation network.

In this way, squeezed phase in MCRT utilizes varying squeeze ratios to strengthen the local semantic feature. The second step of the process called the excitation operation which uses a fully connected layer to capture channel-wise dependencies. MCRT extracts features by fusing the information of four parallel paths including one skip connection as shown in Fig. 3. The enhanced feature using multiple squeeze ratio followed by convolution, batch normalization, and ReLU in three parallel paths are added with input using identity connection. Let the transformation $T:X\mapsto Y$, Where $X\in \mathbb {R^{H\times W\times C}}$, $Y\in \mathbb {R^{H\times W\times C}}$ H, W, and C are the height, width, and depth of the input feature.

Let $X=\left[ x^{1},x^{2}, x^{3}........x^{C} \right]$ be the input of MCRT and $K^{i}=\left[ k_{1}^{i},k_{2}^{i}, k_{3}^{i}.........k_{C}^{i} \right]$ be the filter kernel for first layer convolution operation.

$i=1,2,3$ represents three parallel paths of MCRT, where each having Conv2D, Batch normalization, ReLU, and SE block.

$Y^{i}=\left[ y_{1}^{i},y_{2}^{i}, y_{3}^{i}.........y_{C}^{i} \right]$ represents the output of each convolutional layers of the MCRT, Where Eq. 1) is convolution operation of input with kernel $k_{c}^{i}=\left[ (k_{c}^{i})^{1},(k_{c}^{i})^{2}, (k_{c}^{i})^{3}.......(k_{c}^{i})^{C} \right]$.

1 $$\begin{aligned} y_{c}^{i}=k_{c}^{i}\star X=\sum _{j=1}^{C}\left( k_{c}^{i} \right) ^{j}\star x^{j} \end{aligned}$$

$(k_{c}^{i})^{j}$ is a 2D spatial kernel and therefore represents a single channel of $K_{c}^{i}$. To generate channel-wise statistics, the first step is to squeeze the global spatial information into the channel parameters called channel descriptors. The $c^{th}$ element of $S_{sq}^{i},\,\,Where \,\ S_{sq}^{i}=\left[ s_{1}^{i}, s_{2}^{i}, s_{3}^{i}.......s_{c}^{i} \right] \in \mathbb {R}^{C}$ is denoted as $s_{c}^{i}$ is calculated by shrinking the transformed output *Y* through its spatial dimensions $(H \times W)$ and expressed in Eq. (2).

2 $$\begin{aligned} \begin{aligned} s_{c}^{i}=\frac{1}{\left( H\times W \right) }\sum _{a=1}^{H}\sum _{b=1}^{W}y_{c}^{i}(a,b) \end{aligned} \end{aligned}$$

The second step of the process called the excitation operation which uses a fully connected layer to capture channel-wise dependencies. The excitation operation expressed in Eqs. (3) and (4), uses a simple gating mechanism with a sigmoid activation function where $W_{1}^{i}\in R^{\left( \frac{C}{r^{i}}\times C \right) }$, $W_{2}^{i}\in R^{\left( C\times \frac{C}{r^{i}}\right) }$.

3 $$\begin{aligned} \widetilde{S_{sq}^{i}}= & \sigma \left( W_{2}^{i}ReLU\left( W_{1}^{i}S_{sq}^{i} \right) \right) \end{aligned}$$

4 $$\begin{aligned} \widetilde{y_{c}^{i}}= & \widetilde{s_{sq(c)}^{i}}\,\,\times y_{c} \end{aligned}$$

The Final output of MCRT module as

5 $$\begin{aligned} \widetilde{\widetilde{Y}}=X+\widetilde{Y^{1}}+\widetilde{Y^{2}}+\widetilde{Y^{3}} \end{aligned}$$

The gradient equation for backpropagation to update the weight is defined as

6 $$\begin{aligned} \begin{aligned} \frac{\partial \widetilde{\widetilde{Y}}}{\partial X}=\frac{\partial \left( X+\widetilde{Y^{1}}+\widetilde{Y^{2}}+\widetilde{Y^{3}} \right) }{\partial X}\\ =1+\frac{\partial \widetilde{Y^{1}}}{\partial X}+\frac{\partial \widetilde{Y^{2}}}{\partial X}+\frac{\partial \widetilde{Y^{3}}}{\partial X} \end{aligned} \end{aligned}$$

where $\frac{\partial \widetilde{Y^{1}}}{\partial X}$, $\frac{\partial \widetilde{Y^{2}}}{\partial X}$, $\frac{\partial \widetilde{Y^{3}}}{\partial X}$ represents the gradient of outputs from three parallel paths with respect to the input.

The extracted features from the MCRT block at three different stages within the network are fed to the group convolution deep localization (GCDL) block. GCDL block is composed of three highly efficient sub-sections called strided convolution block, attention mechanism, and aggregated depthwise separable convolution block. GCDL block is intended to improve the generalization power of the proposed CNN model and add more nonlinearity to the representation learned by the previous layer with limited computational complexity.

The term stride defines the step size of the kernel when sliding through the image. The purpose of using strided convolution is to scale each stage output feature into similar dimensions. There are other techniques to do this but this is advantageous in the sense that strided convolution performs pooling as well as convolution operation. In convolution, we have a kernel and that kernel gets multiplied by the image pixel value with a given stride. Since strided convolution performs both pooling and convolution, pooling alters the dimension as per requirement whereas the convolution process has some learnable parameters, which enhance the feature learning capability. Let $X_{1}$, $X_{2}$, and $X_{3}$ are the input feature received from different stages of the network and passed it to three parallel strided convolution layer. The results of these parallel strided convolution are concatenated channel-wise and is expressed in Eq. (7).

7 $$\begin{aligned} \begin{aligned} X_{Concat}=\left( X_{1}*K_{1}^{3\times 3} \right) _{stride=4}\copyright \left( X_{2}*K_{2}^{3\times 3} \right) _{stride=2}\\ \copyright \left( X_{3}*K_{3}^{3\times 3} \right) _{stride=1} \end{aligned} \end{aligned}$$

where $K_{1}^{3\times 3}$, $K_{2}^{3\times 3}$, and $K_{3}^{3\times 3}$ are the kernels used in the strided convolution at three stages, $*$-convolution operation, and $\copyright$-concatenation operation.

The resulting concatenated feature undergone through another separable convolution operation with kernel $K_{4}^{3\times 3}$ to combine the effect of three different stages. Equation (8) represents the output feature vector $\left( X_{out}\right)$ of strided convolution block.

8 $$\begin{aligned} \begin{aligned} X_{out}=\left( X_{Concat}*K_{4}^{3\times 3} \right) _{stride=1} \end{aligned} \end{aligned}$$

Further, $X_{out}$ is passed through ReLU activation, and after that global pooling is carried out to improve the awareness of global information. The obtained feature vector is denoted as $X_{out}^{GP}$ and it is the overall effect of all previous operations.

The $c^{th}$ element of $\left( X_{out}^{GP}\right) , (X_{out}^{GP}\in \mathbb {R}^{C})$, denoted as $\left( X_{out}^{GP}\right) _{c}$ is calculated by shrinking the transformed output through its spatial dimensions $(H \times W)$ and expressed in Eq. (9).

9 $$\begin{aligned} \left( X_{out}^{GP}\right) _{c}=\frac{1}{\left( H\times W \right) }\sum _{a=1}^{H}\sum _{b=1}^{W}(X_{out})_{c}(a,b) \end{aligned}$$

In the attention mechanism^47,48^ the output feature vector of MCRT block-3 is $X_{3}$ gets magnified by the factor $X_{out}^{GP}$. $X_{3}$ is obtained after three subsequent convolution operations of feature space 16, 32, 64, and enriched nuclear feature by the MCRT block-3. The data of $X_{3}$ contains the highest contextual information about the nuclear region of the whole of the network. $X_{out}^{GP}$ has the overall effect of all previous operations. The magnified vector expressed in Eq. (10) is the multiplication of $X_{out}^{GP}$ and $X_{3}$ and it contributes in object localization. Attention mechanism also helps to extract contextual information of granular tumor regions and improve classification accuracy.

10 $$\begin{aligned} X_{AM}=(X_{out}^{GP}\times X_{3}) \end{aligned}$$

## Results and discussion

In this work, we trained existing deep-learning models and evaluated the RenalNet architecture, which demonstrates the potential to classify RCC from kidney histopathology images. Further, the RenalNet architecture reduces the need for extensive computations by incorporating highly optimized modules in the network. The quality metrics and the prediction of the RenalNet model is compared with other classification networks like ShuffleNet, ESPNetV2, BHCNet, Pan-RCC, BreastNet, LiverNet, Vision Transformer, and Hybrid Deep Feature Fusion (HDFF) Network. Five fold cross-validation to analyze how the model is working other than a particular set of data is also presented. A detailed computational complexity analysis of the RenalNet architecture and other reference models is presented. Additionally, the performance of the networks, enhanced through transfer learning using pre-trained weights from the ImageNet dataset, is thoroughly evaluated.

### Comparison with state-of-the-art techniques

**Fig. 4:**
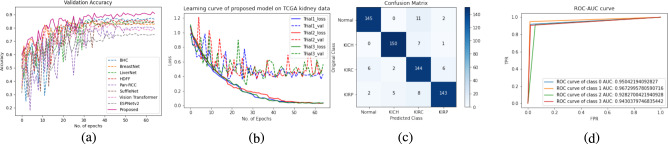
Learning curve of RenalNet on introduced TCGA kidney dataset (**a**) Comparison of validation accuracy of state-of-the-art models (**b**) Training and validation loss of three trials (**c**) Confusion matrix of the best trial (**c**) ROC-AUC of test data for four class classification class-0 (KICH) class-1 (KIRC), class-2 (KIRP), class-3 (Normal).

**Fig. 5:**
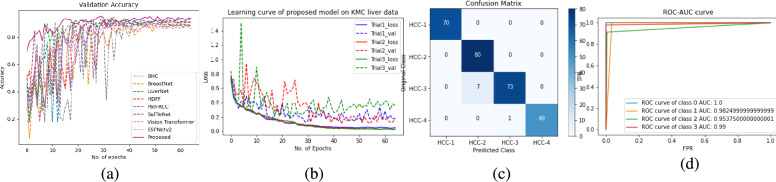
Learning curve of RenalNet on KMC liver dataset (**a**) Comparison of validation accuracy of state-of-the-art models (**b**) Training and validation loss of three trials (**c**) Confusion matrix of the best trial (**c**) ROC-AUC of test data for four class classification class-0 (HCC-1) class-1 (HCC-2), class-2 (HCC-3), class-3 (HCC-4).

**Fig. 6:**
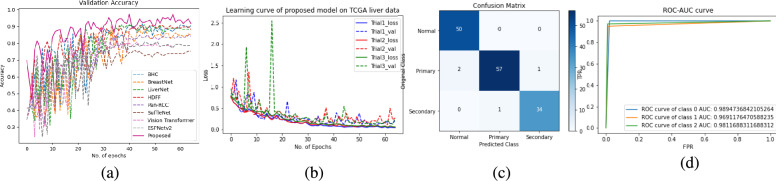
Learning curve of RenalNet on TCGA liver dataset (**a**) Comparison of validation accuracy of state-of-the-art models (**b**) Training and validation loss of three trials (**c**) Confusion matrix of the best trial (**d**) ROC-AUC of test data for three class classification class-0 (Normal) class-1 (Primary), class-2 (Secondary).

**Fig. 7:**
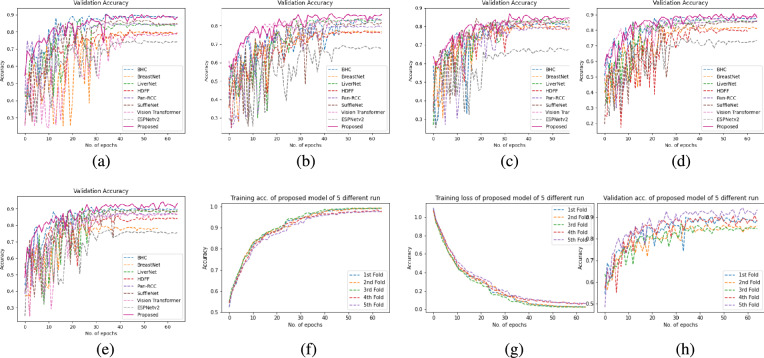
5-Fold cross-validation learning curve of RenalNet on proposed TCGA kidney dataset. (**a**) to (**e**) Comparison of validation accuracy of state-of-the-art models of five different fold (**f**) Training accuracy of five different fold (**g**) Training loss of five different fold (**h**) Comparison of validation accuracy of the proposed model of five different fold.

The results of the proposed model have been compared with the benchmark models ShuffleNet^43^, ESPNetV2^45^, BHCNet^14^, Pan-RCC^5^, BreastNet^11^, LiverNet^17^, Vision Transformer^40,41^, and HDFF^42^. All models were evaluated on three different histopathology datasets: (1) Proposed TCGA kidney dataset (2) KMC Liver dataset (3) TCGA Liver dataset. Our model substantially outperforms the other models in terms of *precision*, *recall*, *F1 score*, and *accuracy*. We experimented with all benchmark models and the proposed model for three trials on each histopathology dataset and evaluated them by randomly initializing the weights in each trial. The reported result in the table is the average of three independent trials of each class. The *F1*, and *accuracy* of the proposed model are 0.9165, and 0.9167 respectively for the proposed TCGA kidney dataset. From the overall *accuracy* point of view for the TCGA kidney dataset the RenalNet model achieved the highest 17% margin on ESPNetv2 while the lowest 4.12 % margin on BHCNet. For KMC Liver dataset, *F1*, and *accuracy* of the proposed model are 0.9740, and 0.9714 respectively. The classwise *Precision*, *Recall*, *F1*, *Accuracy* and overall performance of all benchmark models and proposed models are presented in Tables 4, 5 and Table 6 for TCGA kidney dataset, KMC Liver dataset, and TCGA Liver dataset respectively. Comparison of validation accuracy of state-of-the-art models, validation loss of three trials, confusion matrix of best trial, and ROC-AUC of test data are shown in Figs. 4, 5 and 6 for each dataset respectively. The learning curves indicate the proposed model is working very well for training data as well as validation data. The proposed model shows the best learning behavior and generalizations to work on different types of histopathology data. ROC-AUC plot indicated the measure of the ability of a classifier between different labels. The higher is ROC-AUC value better the performance of the model. From the ROC-AUC viewpoint, our model is best for each dataset. Interquartile length and median is a good measures to check the dispersion of data in any box plot. Figure 7 is the box plot of *Accuracy* of all comparison models. The above box plot indicates that the proposed RenalNet outperforms the other models in all three histopathology datasets.

**Table 4:** Performance metrics comparison of proposed RenalNet with other competitive models on introduced TCGA kidney dataset.

Metrics	Class	ShuffleNet (2018)	ESPNetv2 (2019)	BHCNet (2019)	Pan-RCC (2019)	BreastNet (2020)	LiverNet (2021)	ViT (2023)	HDFF (2024)	RenalNet (Proposed)
Precision	KICH	0.8544	0.7056	0.8818	0.8341	0.8331	0.8788	0.8620	0.8562	**0.9327**
	KIRC	0.8771	0.8620	0.9288	0.8637	0.9063	0.9072	0.7946	0.8698	**0.9477**
	KIRP	0.8236	0.7209	0.8197	0.7983	0.8400	0.8346	0.7622	0.8620	**0.8651**
	Normal	0.8736	0.7172	0.8765	0.8398	0.8497	0.8412	0.7798	0.9006	**0.9244**
	**Overall**	**0.8572**	**0.7514**	**0.8767**	**0.8340**	**0.8573**	**0.8654**	**0.7996**	**0.8722**	**0.9175**
Recall	KICH	0.9029	0.7890	0.8776	0.8438	0.8734	0.8966	0.7911	0.9050	**0.9536**
	KIRC	0.9324	0.8607	0.9388	0.9219	0.9472	0.9240	0.9303	0.9303	**0.9494**
	KIRP	0.7911	0.6371	0.8396	0.7447	0.8101	0.7721	0.6898	0.7911	**0.8881**
	Normal	0.8016	0.6983	0.8460	0.8291	0.7953	0.8607	0.7848	0.8607	**0.8755**
	**Overall**	**0.8571**	**0.7463**	**0.8755**	**0.8349**	**0.8565**	**0.8633**	**0.7990**	**0.8718**	**0.9167**
F1	KICH	0.8778	0.7422	0.8791	0.8386	0.8519	0.8857	0.8250	0.88	**0.9426**
	KIRC	0.9039	0.8608	0.9335	0.8918	0.9258	0.9144	0.8571	0.8990	**0.9484**
	KIRP	0.8066	0.6673	0.8290	0.7703	0.8245	0.7983	0.7242	0.8250	**0.8761**
	Normal	0.8358	0.7061	0.8604	0.8344	0.8203	0.8508	0.7823	0.8802	**0.8992**
	**Overall**	**0.8560**	**0.7441**	**0.8755**	**0.8337**	**0.8556**	**0.8623**	**0.7972**	**0.8711**	**0.9165**
Accuracy	KICH	0.9372	0.8634	0.9393	0.9187	0.9235	0.9425	0.9161	0.9382	**0.9710**
	KIRC	0.9504	0.9303	0.9668	0.9441	0.9620	0.9572	0.9224	0.9477	**0.9741**
	KIRP	0.9051	0.8433	0.9135	0.8892	0.9140	0.9024	0.8686	0.9161	**0.9372**
	Normal	0.9214	0.8554	0.9314	0.9177	0.9135	0.9245	0.8908	0.9414	**0.9509**
	**Overall**	**0.8571**	**0.7463**	**0.8755**	**0.8349**	**0.8565**	**0.8633**	**0.7990**	**0.8718**	**0.9167**

Significant values are in bold.

**Table 5:** Performance metrics comparison of proposed RenalNet architecture with other competitive models on KMC liver dataset.

Metrics	Class	ShuffleNet (2018)	ESPNetv2 (2019)	BHCNet (2019)	Pan-RCC (2019)	BreastNet (2020)	LiverNet (2021)	ViT (2023)	HDFF (2024)	RenalNet (Proposed)
Precision	HCC-1	1	1	0.9950	0.9907	1	1	0.9352	1	**1**
	HCC-2	0.9051	0.8392	0.9364	0.9120	0.8756	0.9350	0.8489	0.8482	**0.9195**
	HCC-3	0.8377	0.8170	0.8562	0.8245	0.8735	0.8834	0.8549	0.889	**0.9865**
	HCC-4	1	1	1	1	1	0.9697	1	1	**1**
	**Overall**	**0.9357**	**0.9140**	**0.9469**	**0.9318**	**0.9373**	**0.9470**	**0.9125**	**0.9445**	**0.9765**
Recall	HCC-1	1	0.9952	0.9619	1	1	1	1	1	**1**
	HCC-2	0.9416	0.8458	0.9083	0.85	0.9083	0.8875	0.8819	0.9443	**1**
	HCC-3	0.9	0.8458	0.9666	0.9125	0.8708	0.9208	0.7944	0.8018	**0.9125**
	HCC-4	0.8133	0.9333	0.8733	0.92	0.9333	0.9733	0.9111	0.871	**0.98**
	**Overall**	**0.9137**	**0.9050**	**0.9275**	**0.9206**	**0.9281**	**0.9454**	**0.8996**	**0.9297**	**0.9731**
F1	HCC-1	1	0.9976	0.9780	0.9953	1	1	0.9986	1	**1**
	HCC-2	0.9225	0.8420	0.9191	0.8796	0.8909	0.9102	0.8626	0.8901	**0.9580**
	HCC-3	0.8672	0.8306	0.9065	0.8651	0.8704	0.9001	0.8202	0.8527	**0.9480**
	HCC-4	0.8969	0.9654	0.9299	0.9573	0.9647	0.9702	0.9574	0.9357	**0.9898**
	**Overall**	**0.9216**	**0.9089**	**0.9334**	**0.9243**	**0.9315**	**0.9451**	**0.9022**	**0.9323**	**0.9740**
Accuracy	HCC-1	1	0.9988	0.9892	0.9976	1	1	0.8723	0.8952	**1**
	HCC-2	0.9547	0.9095	0.9547	0.9333	0.9369	0.95	0.8911	0.9064	**0.975**
	HCC-3	0.9214	0.9011	0.9428	0.9190	0.925	0.9416	0.8613	0.9111	**0.9714**
	HCC-4	0.9666	0.9880	0.9773	0.9857	0.9881	0.9892	0.8956	0.8988	0.9964
	**Overall**	**0.9214**	**0.8988**	**0.9321**	**0.9178**	**0.925**	**0.9404**	**0.8832**	**0.9142**	**0.9714**

Significant values are in bold.

**Table 6:** Performance metrics comparison of proposed RenalNet architecture with other competitive models on TCGA liver dataset.

Metrics	Class	ShuffleNet (2018)	ESPNetv2 (2019)	BHCNet (2019)	Pan-RCC (2019)	BreastNet (2020)	LiverNet (2021)	ViT (2023)	HDFF (2024)	RenalNet (Proposed)
Precision	Normal	0.8938	0.9255	0.9901	0.9450	0.9743	0.9685	0.8996	0.9275	**0.9615**
	Primary	0.8071	0.8425	1	0.7577	0.9326	0.9115	0.8978	0.92	**0.9827**
	Secondary	0.6209	0.6851	0.8464	0.6388	0.7979	0.8976	0.6978	0.9072	**0.9714**
	**Overall**	**0.7739**	**0.8177**	**0.9455**	**0.7805**	**0.9016**	**0.9259**	**0.8318**	**0.9398**	**0.9719**
Recall	Normal	0.9066	0.98	1	1	1	0.9866	0.989	0.9216	**1**
	Primary	0.5777	0.7222	0.8833	0.7666	0.8444	0.9111	0.676	0.8841	**0.95**
	Secondary	0.8666	0.7904	1	0.5714	0.8952	0.8666	0.8947	0.9835	**0.9714**
	**Overall**	**0.7837**	**0.8308**	**0.9611**	**0.7793**	**0.9132**	**0.9214**	**0.8529**	**0.9297**	**0.9738**
F1	Normal	0.8893	0.9515	0.9950	0.9713	0.9869	0.9770	0.9423	0.9166	**0.9804**
	Primary	0.6516	0.7762	0.9377	0.7617	0.8862	0.9098	0.7709	0.9226	**0.9661**
	Secondary	0.7210	0.7332	0.9160	0.6031	0.8434	0.8793	0.784	0.938	**0.9714**
	**Overall**	**0.7540**	**0.8203**	**0.9495**	**0.7787**	**0.9055**	**0.9221**	**0.8324**	**0.9258**	**0.9726**
Accuracy	Normal	0.9241	0.9655	0.9965	0.9793	0.9908	0.9839	0.9745	0.9918	**0.9862**
	Primary	0.7609	0.8275	0.9517	0.8022	0.9103	0.9264	0.8365	0.9223	**0.9724**
	Secondary	0.8367	0.8620	0.9551	0.8183	0.9195	0.9425	0.871	0.92	**0.9862**
	**Overall**	**0.7609**	**0.8275**	**0.9517**	**0.8000**	**0.9103**	**0.9264**	**0.8365**	**0.9203**	**0.9724**

Significant values are in bold.

### 5-fold cross validation

**Fig. 8:**
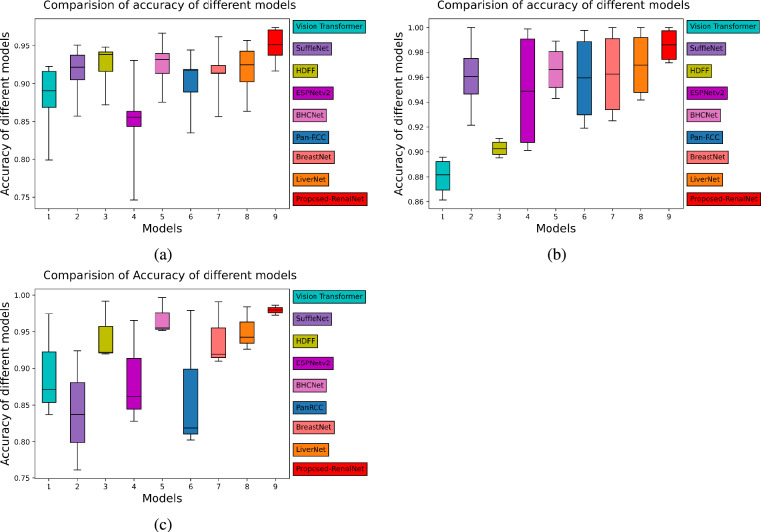
Box Plot: (**a**–**c**) Accuracy of different models for new TCGA kidney dataset, KMC Liver dataset, TCGA Liver dataset.

 In the proposed dataset, the training set and test set are slide-wise separated such that the test set was completely unseen for the model. However, it is possible that some cases in the test set have the same content as in the training set. In this way, the model might not produce the true result. To avoid such issues model was tested using 5-fold cross-validation. This allows us to analyze how the model is working apart from a particular set of test data. To do so, 5 folds of 880 images of each class were created to carry out 5-fold cross-validation. For the first-run fold-2 to fold-5 is used to train the model while fold-1 is to test the model. In the second run fold-1, fold-3 to fold-5 are used to train the model while fold-2 is to test the model. We repeat the process till the last fold where fold-5 is for testing and the remaining fold is for training the model. The average results of all five runs for the proposed model and all benchmark models are included in Table 7. The proposed model attains almost similar results given in Table 4. BreastNet^11^, ViT^41^ and ESPNetV2^45^ show considerable variations in the result of Tables 4 and 7. The Fig. 8 shows the learning curves of the proposed model for the 5-fold cross-validation. Validation accuracy of all 5-folds of the proposed model and reference models, and validation accuracy of five different runs of the proposed model shows almost the same learning behavior, this suggests that the proposed model is generalized independent of the dataset.

**Table 7:** 5-Fold cross validation average quality metrics comparison of proposed RenalNet with other competitive models on introduced kidney dataset.

Metrics	Class	ShuffleNet (2018)	ESPNetv2 (2019)	BHCNet (2019)	Pan-RCC (2019)	BreastNet (2020)	LiverNet (2021)	ViT (2023)	HDFF (2024)	RenalNet (Proposed)
Precision	KICH	0.8740	0.6990	0.8820	0.8432	0.8174	0.8707	0.892	0.8412	**0.9116**
	KIRC	0.8602	0.7903	0.8850	0.8793	0.8695	0.8983	0.8246	0.8548	**0.9005**
	KIRP	0.8019	0.7050	0.8223	0.7732	0.8041	0.8333	0.7922	0.847	**0.8748**
	Normal	0.8961	0.7344	0.8839	0.8496	0.7968	0.8911	0.8098	0.8856	**0.9229**
	**Overall**	**0.8581**	**0.7322**	**0.8683**	**0.8363**	**0.8219**	**0.8733**	**0.8296**	**0.8572**	**0.9024**
Recall	KICH	0.8845	0.81	0.8818	0.8490	0.8545	0.8909	0.8211	0.8900	**0.9063**
	KIRC	0.8963	0.8054	0.9045	0.8572	0.8490	0.9054	0.9603	0.9153	**0.9373**
	KIRP	0.8045	0.6481	0.81	0.7490	0.7563	0.8045	0.7198	0.7761	**0.8491**
	Normal	0.8372	0.6527	0.8718	0.8773	0.79	0.8818	0.8148	0.8456	**0.9118**
	**Overall**	**0.8557**	**0.7291**	**0.8670**	**0.8332**	**0.8125**	**0.8706**	**0.829**	**0.8568**	**0.9011**
F1	KICH	0.8777	0.7500	0.8814	0.8438	0.8310	0.8785	0.855	0.865	**0.9077**
	KIRC	0.8766	0.7971	0.8929	0.8656	0.8523	0.8989	0.8871	0.884	**0.9172**
	KIRP	0.8025	0.6720	0.8154	0.7605	0.7758	0.8179	0.7542	0.81	**0.8614**
	Normal	0.8649	0.6889	0.8777	0.8619	0.7911	0.8856	0.8123	0.8652	**0.9169**
	**Overall**	**0.8554**	**0.7270**	**0.8668**	**0.8329**	**0.8125**	**0.8702**	**0.8272**	**0.8561**	**0.9008**
Accuracy	KICH	0.9384	0.8647	0.9404	0.9216	0.9120	0.9388	0.9461	0.9232	**0.9541**
	KIRC	0.9366	0.8977	0.9457	0.9336	0.9263	0.9486	0.9524	0.9327	**0.9572**
	KIRP	0.9016	0.8418	0.9086	0.8818	0.8907	0.9104	0.8986	0.9011	**0.9320**
	Normal	0.9347	0.8538	0.9393	0.9293	0.8959	0.9434	0.9208	0.9264	**0.9588**
	**Overall**	**0.8557**	**0.7291**	**0.8670**	**0.8332**	**0.8125**	**0.8706**	**0.829**	**0.8568**	**0.9011**

Significant values are in bold.

### Transfer learning

The performance of networks leveraged by transfer learning of pre-trained weights from the ImageNet dataset is shown in Table 8. Here all the layers are frozen except very few layers at the top are trained for 30 epochs. The quality metrics and the prediction of the proposed RenalNet model trained from scratch are compared with other classification networks like VGG-16, ResNet-50, ResNet-150, DenseNet, InceptionNet, InceptionResNetV2, MobileNetV2, and NasNet, which is using pre-trained weights from the ImageNet dataset. From the overall *accuracy* point of view for the TCGA kidney dataset, the RenalNet model achieved the highest margin of 11.93% on NasNet while the lowest margin of 4.65 % on DenseNet. Among all examinations, CNN models like BHCNet, LiverNet, BreastNet, and the proposed RenalNet achieve acceptable results without transfer learning. The proposed RenalNet, trained from scratch, achieved the highest accuracy for renal cell carcinoma (RCC) detection and two additional histopathology datasets.

**Table 8:** Performance metrics comparison of proposed RenalNet trained from scratch with other models using pre-trained weights from the ImageNet dataset (Proposed TCGA kidney dataset).

Metrics	Class	VGG-16	ResNet-50	ResNet-150	DenseNet	Inception	Inception-ResnetV2	Mobile NetV2	NasNet	RenalNet (Trained from scratch)
Precision	KICH	0.9231	0.7964	0.7964	0.875	0.8562	0.8431	0.8259	0.8258	**0.9327**
	KIRC	0.9068	0.8742	0.8650	0.9295	0.8152	0.8820	0.9032	0.8312	**0.9477**
	KIRP	0.7391	0.7814	0.7615	0.8072	0.7549	0.7654	0.7559	0.7362	**0.8651**
	Normal	0.8471	0.8451	0.8543	0.8734	0.7953	0.8525	0.8506	0.7987	**0.9244**
	**Overall**	**0.8540**	**0.8243**	**0.8193**	**0.8712**	**0.8054**	**0.8358**	**0.8339**	**0.7980**	**0.9175**
Recall	KICH	0.7595	0.8417	0.8417	0.8417	0.8291	0.8164	0.8101	0.8101	**0.9536**
	KIRC	0.9240	0.8797	0.8924	0.9177	0.8101	0.8987	0.8861	0.8417	**0.9494**
	KIRP	0.8607	0.7468	0.7278	0.8481	0.7215	0.7848	0.8038	0.7595	**0.8881**
	Normal	0.8417	0.8291	0.8164	0.8734	0.8607	0.8417	0.8291	0.7785	**0.8755**
	**Overall**	**0.8465**	**0.8243**	**0.8196**	**0.8702**	**0.8053**	**0.8354**	**0.8322**	**0.7975**	**0.9167**
F1 Score	KICH	0.8333	0.8184	0.8185	0.8581	0.8424	0.8295	0.8179	0.8179	**0.9426**
	KIRC	0.9154	0.8770	0.8785	0.9235	0.8127	0.8903	0.8945	0.8365	**0.9484**
	KIRP	0.7953	0.7637	0.7443	0.8271	0.7378	0.7750	0.7791	0.7476	**0.8761**
	Normal	0.8444	0.8370	0.8349	0.8734	0.8267	0.8471	0.8397	0.7884	**0.8992**
	**Overall**	**0.8471**	**0.8241**	**0.8191**	**0.8705**	**0.8049**	**0.8354**	**0.8328**	**0.7976**	**0.9165**
Accuracy	KICH	0.9240	0.9066	0.9066	0.9303	0.9224	0.9161	0.9098	0.9098	**0.9710**
	KIRC	0.9573	0.9382	0.9383	0.9620	0.9066	0.9446	0.9477	0.9177	**0.9741**
	KIRP	0.8892	0.8844	0.875	0.9113	0.8718	0.8861	0.8860	0.8718	**0.9372**
	Normal	0.9225	0.9193	0.9193	0.9367	0.9098	0.9240	0.9208	0.8955	**0.9509**
	**Overall**	**0.8465**	**0.8243**	**0.8196**	**0.8702**	**0.8053**	**0.8354**	**0.8322**	**0.7974**	**0.9167**

Significant values are in bold.

**Fig. 9:**
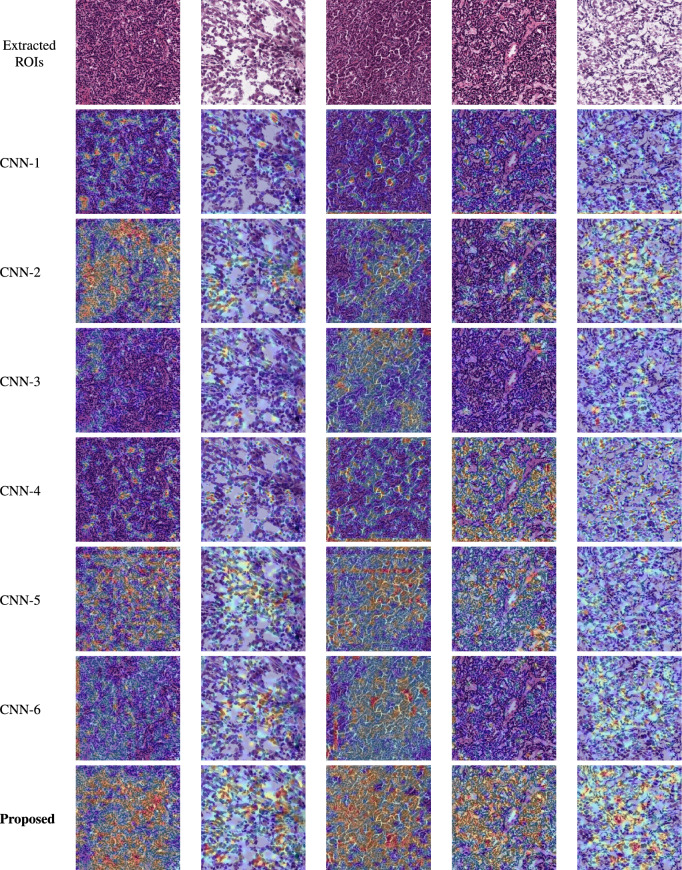
Visual performance comparison of different CNN variations using activation maps. (Red: Very high probability score regions, Orange: Medium probability score regions, Light blue: Low probability score regions).

### Computational complexity analysis

From the computational complexity viewpoint, the proposed RenalNet model showed effectiveness in comparison with other competitive models. Computational complexity is expressed in terms of the total trainable parameters and floating-point operations (FLOPs) shown in Table 9. The proposed model utilizes the least number of trainable parameters with respect to any other comparable models. ESPNetV2^45^ has the least number of FLOPs which is 0.527 billion, while the proposed model utilizes 2.71 billion FLOPs. Training and prediction time are also good to measure the complexity of a model. We have trained the proposed model and other benchmark models for 65 epochs in the same training and test environment. These values are given in Table 9. The time required for training and prediction of the proposed model is 1.227 hrs and 12.73 sec respectively, which is minimum compared to any other model.

**Table 9:** Computational complexity comparison of architectures.

Model	Parameters (M = $10^6$)	FLOPs (G = $10^9$)	Training Time (Hours)	Prediction Time in Second (Test Set)
ShuffleNet(2018)	23.8230 M	6.69 G	2.058	15.77
ESPNetV2 (2019)	3.9134 M	0.527 G	1.986	21.71
BHCNet(2019)	0.3020 M	4.27 G	1.986	20.95
Pan-RCC(2019)	23.5960 M	7.75 G	1.462	16.02
BreastNet(2020)	0.6061 M	5.68 G	1.535	20.31
LiverNet(2021)	0.5740 M	3.72 G	2.112	26.66
Vision Transformer(2023)	33.5 M	7.2 G	3.20	27.50
HDFF(2024)	34.689 M	87.564 G	3.339	31.83
**RenalNet**	**0.2131 M**	**2.71 G**	**1.227**	**12.73**

Significant values are in bold.

Ablation study The proposed RenalNet is composed of two main modules. (I) Multiple channel residual transformation (MCRT). (II) Group convolution deep localization (GCDL) network. GCDL block contains three highly efficient sub-modules called strided convolution block, attention mechanism, and aggregated depthwise separable convolution block. To demonstrate the effectiveness of the proposed RenalNet, we measured the strength of individual modules as well as some combination of these by detaching the main module from the proposed RenalNet. All these different versions of the network were trained on the same environment and performance was compared with the proposed RenalNet, where all these important modules were jointly worked. The overall test accuracy, and other quality metrics of different versions are listed in Table 10. A comparison of the computational complexity of different variations was expressed in terms of the total number of parameters and floating-point operations (FLOPs). The visual performance of each variation is analyzed in Fig 9, by using activation maps which show how accurately the model separates the malignant tissue regions from the background.

**Table 10:** Overall quality comparison of eight variants of proposed RenalNet *MCRT*: Multiple channel residual transformation, *GCDL*: Group convolution deep localization, *ADSC*:Aggregated depthwise separable convolution, *AM*: Attention mechanism, SE with excitation ratio (R)= 2, 4, 8.

Model Variations	MCRT	GCDL	ADSC	AM	R=2	R=4	R=8	F1 Score	ACC	Parmeters	FLOPs (G = $10^9$)
CNN-1	$\checkmark$	$\times$	$\times$	$\times$	$\checkmark$	$\checkmark$	$\checkmark$	0.8924	0.8924	182,886	2.58G
CNN-2	$\times$	$\checkmark$	$\checkmark$	$\checkmark$	$\times$	$\times$	$\times$	0.8801	0.8813	56,500	0.609G
CNN-3	$\checkmark$	$\times$	$\checkmark$	$\times$	$\checkmark$	$\checkmark$	$\checkmark$	0.8969	0.8971	208,582	2.65G
CNN-4	$\checkmark$	$\times$	$\times$	$\checkmark$	$\checkmark$	$\checkmark$	$\checkmark$	0.8957	0.8955	202,038	2.7G
CNN-5	$\checkmark$	$\checkmark$	$\checkmark$	$\checkmark$	$\checkmark$	$\times$	$\times$	0.8994	0.9003	219,964	2.71G
CNN-6	$\checkmark$	$\checkmark$	$\checkmark$	$\checkmark$	$\times$	$\checkmark$	$\times$	0.8981	0.8987	211,816	2.71G
CNN-7	$\checkmark$	$\checkmark$	$\checkmark$	$\checkmark$	$\times$	$\times$	$\checkmark$	0.9093	0.9098	207,742	2.71G
**Proposed**	$\checkmark$	$\checkmark$	$\checkmark$	$\checkmark$	$\checkmark$	$\checkmark$	$\checkmark$	**0.9165**	**0.9167**	**213,174**	**2.71G**

Significant values are in bold.

### Comparision of activation maps of different variations

The classification performance depends on the probability score assigned to the relevant region. CNN-1, where the complete GCDL block is detached from the proposed network, assigns a high probability score for most of the nuclear regions but also assigns a low probability value to background pixels in some cases. CNN-3 and CNN-4 avoid such conditions. CNN-3 measures the strength of GCDL with aggregated depth-wise separable convolution block alone while CNN-4 evaluates GCDL capability with attention mechanism part only. CNN-4 assigns a very high probability to granular tumor regions compared to any other variations but images that have larger nuclear regions are not detected properly. This shortcoming with CNN-4 is somewhat solved with CNN-3. The proposed method utilizes the combined effect of CNN-1, CNN-3, and CNN-4, such that it is helpful in avoiding false-positive cases. In CNN-2 high probability values are less in number but help to reduce false-positive cases to some extent. By using the same channel ratio in three parallel SE blocks as in CNN-5, CNN-6, and CNN-7, as the ratio increases, the true positive cases increase, at the same time it assigns low probability values for background regions. The proposed model uses multiple channels ratio, assigns high probability values to nuclear regions, segregates tissue regions from the background properly and, enhances the level of predictions.

### Without Group convolution deep localization

This variant of the proposed model attains an accuracy of 89.24%, and an F1 score of 89.24%. GCDL block contains three efficient sub-sections called strided convolution block, attention mechanism, and aggregated depth-wise separable convolution block. The strength of each sub-module in GCDL is also measured. We observed a performance drop after detaching the GCDL block and its constituent parts from the proposed model. Very small variations in computational complexity were observed however its contribution to the proposed model cannot be ignored. The produced activation map of different variations indicated that the introduced GCDL block was useful in improving the generalization power of the proposed CNN model to learn and adapt other histopathology data.

### Without multiple channel residual transformation

We placed a novel CNN module called MCRT composed of three parallel branches of the convolutional unit, squeeze and excitation (SE) channel attention unit, and one skip connection. The squeezed phase in three SE units utilizes different squeeze ratios to strengthen the local semantic feature. This block contributes maximally to the proposed RenalNet model and completes the classification process. Without MCRT, results in a drop of 3.54 %, and 3.64% in accuracy, and F1-score respectively from the proposed model. Table 10 and Fig. 9 shows that the proposed RenalNet is the best-performing combination among all modifications made in the base model. This ablation study helps us to choose the best-performing combination among all variations. The two important modules together make the proposed RenalNet very effective in feature extraction with minimum computational complexity.

## Conclusion

This paper proposed a fast and accurate deep learning (DL) based automated system for the detection of renal cell carcinoma (RCC) from kidney histopathology images. The proposed RenalNet model experimented with four classes (Normal, KIRP, KIRC, and KICH) classification of patches of WSIs of TCGA kidney dataset. Extraction of a class-specific representative set of features was possible due to the utilization of optimized transformation modules at three different resolutions within the network. The results of the proposed model have been compared with the existing DL models trained from scratch as well as networks leveraged by transfer learning of pre-trained weights. During our experimentation, the proposed network achieved an *accuracy* of 0.9167, and *F1-Score* 0.9165 on the introduced TCGA kidney dataset, which was the highest among all competitive models. The experimental result indicated that our proposed model gave better results compared to the most recent existing DL models in terms of quality metrics, computational complexity, and training and prediction time. Our future work will focus on the hardware implementation of the RenalNet model for RCC detection from kidney histopathology images.

## Data Availability

The data and code are available at https://github.com/chanchalnitk/RCC_Classification
